# Motion of left atrial appendage as a determinant of thrombus formation in patients with a low CHADS2 score receiving warfarin for persistent nonvalvular atrial fibrillation

**DOI:** 10.1186/1476-7120-10-50

**Published:** 2012-12-27

**Authors:** Koji Ono, Makoto Iwama, Masanori Kawasaki, Ryuhei Tanaka, Takatomo Watanabe, Noriyuki Onishi, Shunichiro Warita, Tai Kojima, Takashi Kato, Yoshiaki Goto, Masazumi Arai, Kazuhiko Nishigaki, Genzou Takemura, Toshiyuki Noda, Sachiro Watanabe, Shinya Minatoguchi

**Affiliations:** 1 Department of Cardiology, Gifu University Graduate School of Medicine, 1-1 Yanagido, Gifu, 501-1194, Japan

**Keywords:** Atrial fibrillation, Left atrial appendage, Thrombus, Transesophageal echocardiography

## Abstract

**Background:**

The aim of this study was to define the independent determinants of left atrial appendage (LAA) thrombus among various echocardiographic parameters measured by Velocity Vector Imaging (VVI) in patients with nonvalvular atrial fibrillation (AF) receiving warfarin, particularly in patients with a low CHADS2 score.

**Methods:**

LAA emptying fraction (EF) and LAA peak longitudinal strain were measured by VVI using transesophageal echocardiography in 260 consecutive patients with nonvalvular persistent AF receiving warfarin. The patients were divided into two groups according to the presence (n=43) or absence (n=217) of LAA thrombus. Moreover, the patients within each group were further divided into subgroups according to a CHADS2 score ≤1.

**Results:**

Multivariate logistic regression analysis showed that LAAEF was an independent determinant of LAA thrombus in the subgroup of 140 with a low CHADS2 score. Receiver operating characteristics curve analysis showed that an LAAEF of 21% was the optimal cutoff value for predicting LAA thrombus.

**Conclusions:**

LAA thrombus formation depended on LAA contractility. AF patients with reduced LAA contractile fraction (LAAEF ≤21%) require strong anticoagulant therapy to avoid thromboembolic events regardless of a low CHADS2 score (≤1).

## 

Left atrial appendage (LAA) thrombus in patients with atrial fibrillation (AF) is a high risk factor of cardiogenic thromboembolism, and causes stroke at the rate of 1.5 - 3.4% per year even in patients receiving warfarin [1-3]. Establishment of optimal risk stratification and therapeutic strategies is the best hope for decreasing the burden of AF-related thromboembolism. The international normalized ratio of prothrombin time (PT-INR) is used as an index for optimal anticoagulation therapy, and the CHADS2 (congestive heart failure, hypertension, age ≥75 years, diabetes mellitus, and prior stroke or transient ischemic attack) score is widely used for risk stratification of thrombus in clinical practice [4,5]. However, it was reported that some AF patients that have PT-INR within the therapeutic range or a low CHADS2 score still suffer from thromboembolism [6].

As for ultrasound parameters, spontaneous echo contrast (SEC) and LAA peak emptying flow velocity (LAAPV) have been proposed as echocardiographic predictors of LAA thrombus [7,8]. However, the optimal cutoff values of ultrasound parameters to predict incidence of LAA thrombus with high sensitivity and specificity have not been established. Velocity Vector Imaging (VVI) has been recently developed based on speckle tracking to evaluate left ventricular function, and this method can be applied to evaluate left atrial (LA) and LAA contractile fraction [9-11]. Thus, the aim of this study was to define the independent determinants of LAA thrombus among various echocardiographic parameters measured by VVI in patients with nonvalvular AF receiving warfarin, particularly in patients with a low CHADS2 score.

## Methods

### Study protocol

We performed transthoracic echocardiography (TTE) and transesopageal echocardiography (TEE) in 300 consecutive patients with warfarin therapy that had persistent AF for more than one month to evaluate intracardiac thrombus, the severity of aortic or mitral valve stenosis or regurgitation or congenital heart disease. Persistent AF was documented electrocardiographically by Holter monitor. There were 37 patients excluded from the study because of mitral stenosis or moderate-to-severe mitral regurgitation or previous mitral valve operation. Three patients were excluded because of poor echocardiographic recordings. The final analysis included 260 patients with nonvalvular persistent AF that were already receiving warfarin. We divided those patients into two groups according to the presence or absence of LAA thrombus. Moreover, each group was divided into subgroups according to a CHADS2 score ≤1 (low CHADS2 subgroup with LAA thrombus and without LAA thrombus) [5]. All patients were on warfarin as an anticoagulant to prevent thromboembolism according to the Japanese Circulation Society guidelines for pharmacotherapy of AF [12]. The PT-INR in all patients was measured one month before and at the time of TTE and TEE. The average of the two PT-INR measurements was used for analysis. LAA thrombus was defined as an echo-dense mass of more than 2 mm in diameter attached to the LAA wall that could be distinguished from the surrounding endocardium or pectinate muscles. The experimental protocol was approved by the ethics committee of our institution and informed consent was obtained from all patients.

### Echocardiography

**Figure 1:**
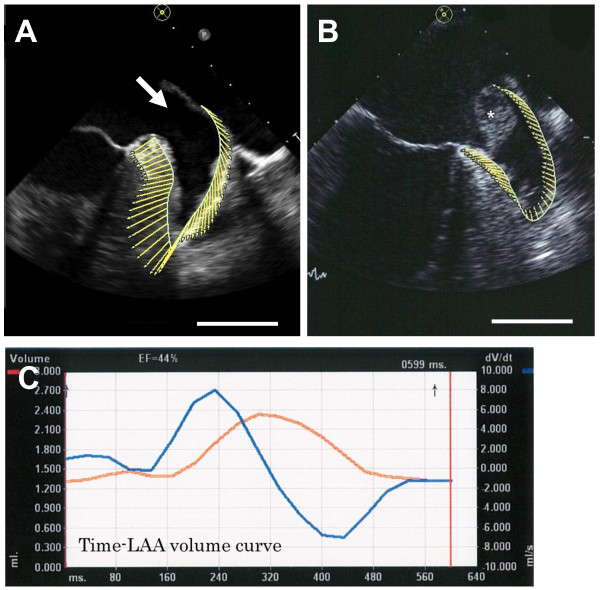
**Representative images of the left atrial appendage, and time-volume curve and time-strain curves constructed by velocity vector imaging in patients with atrial fibrillation.** (**A**) Left atrial appendage (LAA) of a patient without thrombus. (**B**) LAA of a patient with thrombus. (**C**) Time-volume curve is shown as an orange line. Time-dv/dt curve is shown as a blue line. *thrombus. Bar=1 cm.

**Figure 2:**
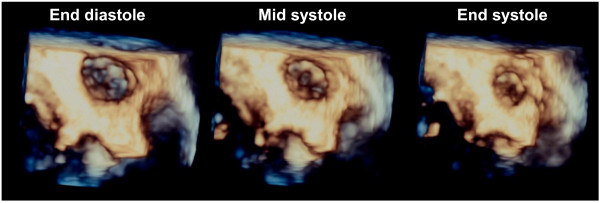
**Three-dimensional view of the left atrial appendage at end diastole, mid systole and end systole from a site indicated by the white arrow in (A) in Figure**1**.**

TTE and TEE were performed using an ACUSON Sequoia 512 ultrasound system (Siemens, Mountain View, CA) to evaluate LAA structure and contractile fraction. We examined the differences in echocardiographic parameters between patients with and without LAA thrombus. The parameters were obtained according to the standards of the American Society of Echocardiography [13,14]. For TEE, a 4-7 MHz multi-plane transducer with a 7.4 mm diameter pediatric probe was used to diminish patient discomfort as previously described [15]. A time-LAA volume curve and a time-LAA strain curve can be automatically and promptly provided during a single cardiac cycle by VVI with feature-tracking echocardiography using off-line software (Syngo Velocity Vector Imaging, Siemens, Mountain View, CA) (Figure 1). We measured LAA volume by two-dimensional TEE three times at three different angles (50, 70 and 90 degrees) and used average values for further analysis to improve the accuracy of the LAA volume measurement, as we previously reported in detail [11]. Average values of three cardiac cycles [(maximum volume – minimum volume)/maximum volume × 100] were used as LAA emptying fraction (EF). LAA volumes were determined using Simpson’s method. Although an autopsy study reported that the LAA is usually a multilobed structure [16], Cardiac computed tomography or magnetic resonance tomography revealed that in 97% of patients, the LAA in vivo was shaped like a cactus (30%), chicken wing (48%) or windsock (19%) [6]. Since three-dimensional TEE showed that the shape of LAA was round throughout the cardiac cycle in the present study, it was considered reasonable to measure LAA volume using the disk summation method (Figure 2). Furthermore, LAAPV, SEC score (grade 0 - 4), LA dimension (LAD) and left ventricular (LV) ejection fraction were measured by the same methods as previously reported [5,11,17]. Excellent reproducibility and reliability of the VVI measurements were demonstrated in previous studies [9-11].

### Statistical analyses

Data for continuous variables were expressed as the mean ± one standard deviation. Categorical data were summarized as percentages and compared using a Chi-square test or a Fisher’s exact test as appropriate. Comparisons of echocardiographic parameters between main groups and between subgroups were performed by an unpaired Student’s *t*-test. Comparisons of SEC, CHADS2 score between groups were performed by a Mann–Whitney *U*-test. The optimal cutoff values for the determination of sensitivity and specificity of the echocardiographic parameters to determine LAA thrombus were obtained from receiver operating characteristic (ROC) curve analysis. Multivariate logistic regression analysis was performed to identify the independent determinants of LAA thrombus. Multivariate logistic regression analysis was performed using clinical variables with a p value ≤0.10 in univariate analysis to determine the independent predictors of LAA thrombus. A p-value <0.05 was considered significant. Statistical analyses were performed using Stat View version 5.0 (SAS Institute Inc, Cary, NC, USA).

## Results

### Patient characteristics

Of the 260 patients with persistent AF included in the present study, 43 patients had LAA thrombus and 217 did not have LAA thrombus. The clinical characteristics of all patients are listed in Table 1. In the patients with a low CHADS2 score (≤1), 15 patients had LAA thrombus and 125 patients did not have LAA thrombus. There were no significant differences in PT-INR, gender, age, history of smoking, history of diabetes mellitus, history of hypertension and duration of AF between the groups with and without LAA thrombus. The target PT-INR levels were 1.6 - 2.6 for patients aged ≥70 years old and 2.0 - 3.0 for patients aged <70 years old, according to the guideline of the Japanese Circulation Society. The target PT-INR was achieved in 31 (73%) of the 43 patients with LAA thrombus, and in 132 (61%) of the 217 patients without LA thrombus. There was no significant difference in the number of patients that achieved the target PT-INR between the two groups (p = 0.16). The target PT-INR was also achieved in 74% of 109 patients aged ≥70 years old and 50% of 151 patients aged <70 years old. The target PT-INR was achieved in a greater percentage of the patients aged ≥70 years old than in the patients aged <70 years old (p <0.001).

**Table 1:** Patient characteristics and echocardiographic parameters in 260 persistent AF patients

	**AF without thrombus**	**AF with thrombus**	**p-value**
	**(n = 217)**	**(n = 43)**	
Clinical parameters			
Age (year)	65.9 ± 10.5	68.9 ± 9.7	0.08
Male, n (%)	165 (76)	35 (81)	0.41
Smoking, n (%)	51 (23)	10 (23)	0.98
Diabetes Mellitus, n (%)	45 (21)	13 (30)	0.17
Hypertension, n (%)	128 (59)	31 (72)	0.10
Dyslipidemia, n (%)	59 (27)	7 (16)	0.13
Prior stroke, n (%)	25 (12)	11 (26)	0.015
AF duration (year)	5.9 ± 5.8	7.1 ± 5.9	0.22
PT-INR	1.89 ± 0.47	1.91 ± 0.44	0.81
PT-INR met guideline	132 (61)	31 (72)	0.16
CHADS2 score	1 (1 – 2)	2 (1 – 3)	0.003
Echocardiographic parameters			
LVEF (%)	57.7 ± 9.2	55.7 ± 9.0	0.19
LAD (mm)	46.7 ± 6.5	49.0 ± 6.1	0.032
Maximum LAA volume (ml)	10.6 ± 4.7	17.4 ± 9.9	<0.001
Minimum LAA volume (ml)	7.4 ± 3.8	14.2 ± 8.4	<0.001
SEC score (0-4 grade)	1 (1 – 1)	1 (1 – 2)	<0.001
LAAPV (cm/sec)	29.6 ± 11.4	21.2 ± 6.6	<0.001
LAA peak longitudinal strain	8.2 ± 5.0	2.7 ± 1.9	<0.001
LAAEF (%)	31.3 ± 10.5	18.5 ± 4.2	<0.001

Numerical data are expressed as the mean ± one standard deviation. Non-parametric data are expressed as the median (interquartile range). AF: atrial fibrillation. PT-INR: prothrombin-international normalized ratio. LAD: left atrial dimension. LVEF: left ventricular ejection fraction. SEC: spontaneous echo contrast. LAAPV: left atrial appendage peak flow velocity. LAAEF: left atrial appendage emptying fraction.

### Echocardiographic parameters

It took approximately one minute (60 ± 10 seconds) to obtain LAAEF and LAA longitudinal strain by VVI. The LAAPV in patients with LAA thrombus was decreased compared with that in patients without LAA thrombus (Table 1). The LAAEF and LAA peak longitudinal strain in patients with LAA thrombus were significantly reduced compared with the corresponding values in patients without LAA thrombus (Table 1). The LAAEF and LAA peak longitudinal strain in the two subgroup with LAA thrombus and a low CHADS2 (Table 2) score were also reduced compared with the corresponding values in the two subgroups without LAA thrombus and a low CHADS2 score.

**Table 2:** Patient characteristics and echocardiographic parameters in 140 persistent AF patients with a low CHADS2 score (≤1)

	**AF without thrombus**	**AF with thrombus**	**p-value**
	**(n = 125)**	**(n = 15)**	
Clinical parameters			
Age (year)	62.1 ± 9.6	65.3 ± 9.0	0.23
Male, n (%)	93 (74)	12 (80)	0.64
Smoking, n (%)	21 (17)	5 (33)	0.16
Diabetes Mellitus, n (%)	9 (7)	1 (7)	>0.99
Hypertension, n (%)	56 (45)	10 (67)	0.11
Dyslipidemia, n (%)	27 (21)	1 (7)	0.30
CHADS2 score = 1	88 (71)	14 (93)	0.059
AF duration (year)	5.9 ± 5.7	6.3 ± 3.4	0.83
PT-INR	1.89 ± 0.49	1.84 ± 0.35	0.65
PT-INR met guideline, n (%)	68 (54)	11 (73)	0.18
Echocardiographic parameters			
LVEF (%)	59.1 ± 6.6	57.3 ± 4.8	0.29
LAD (mm)	46.0 ± 6.7	49.5 ± 7.5	0.064
Maximum LAA volume (ml)	10.2 ± 4.7	17.9 ± 8.1	<0.001
Minimum LAA volume (ml)	7.0 ± 3.8	14.8 ± 7.1	<0.001
SEC score (0-4 grade)	1 (1 – 1)	1 (1 – 2)	0.047
LAAPV (cm/sec)	31.9 ± 12.2	23.0 ± 8.3	0.007
LAA peak longitudinal strain	9.3 ± 5.6	2.5 ± 1.5	<0.001
LAAEF (%)	33.5 ± 10.7	17.9 ± 3.6	<0.001

Abbreviations as in Table 1.

### Determinants of LAA thrombus

We performed multivariate analysis using three models to identify the independent determinants of LAA thrombus in the patients with a low CHADS2 score: Model 1 included only clinical variables with a p value ≤0.10 in univariate analysis: Model 2 included conventional echocardiographic parameters (SEC and LAAPV): and Model 3 included conventional and VVI echocardiographic parameters (SEC, LAAPV and LAAEF). However, LAA peak longitudinal strain was excluded from Model 3 because there was a strong correlation between LAA peak longitudinal strain and LAAEF (r = 0.80, p < 0.001). The multivariate logistic regression analysis showed that LAAEF was an independent determinant of LAA thrombus in the patients with a low CHADS2 score (≤1) (Table 3).

**Table 3:** Age adjusted multivariate logistic regression analysis for LAA thrombus 140 persistent AF patients with a low CHADS2 score (≤1)

	**Odds ratio**	**95% Confidence interval**	**p-value**
Variables (Model 1)			
Hypertension	1.771	0.851 – 3.686	0.13
Prior stroke	2.409	1.037 – 5.597	0.041
Variables (Model 2)			
SEC	1.440	0.730 – 2.843	0.29
LAAPV (cm/sec)	0.931	0.871 – 0.996	0.038
Variables (Model 3)			
SEC	0.864	0.335 – 2.226	0.76
LAAPV (cm/sec)	0.954	0.862 – 1.055	0.36
LAAEF (%)	0.570	0.415 – 0.783	<0.001

Abbreviations as in Table 1.

**Figure 3:**
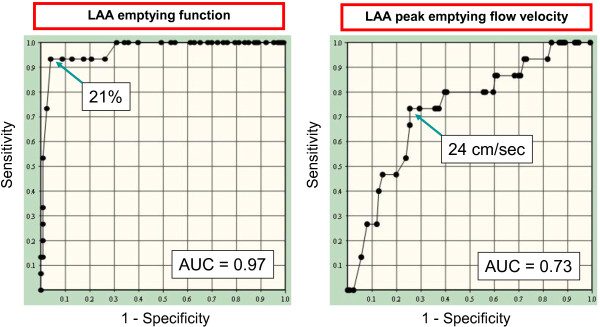
**Receiver operating characteristic curves for the prediction of left atrial appendage thrombus in 140 persistent atrial fibrillation patients with low CHADS2 score (≤1).** LAA: left atrial appendage, AUC: Area under the curve.

Using an LAAEF of 21% as the cutoff value, ROC curve analysis showed that the sensitivity was 93%, the specificity was 96% and the area under the curve (AUC) was 0.97. Using an LAAPV cutoff value of 24 cm/sec, the sensitivity was 73%, the specificity was 75% and the AUC was 0.73 (Figure 3). The sensitivities, specificities, positive predictive values and negative predictive values at the optimal cutoff values are listed in Table 4.

**Table 4:** Accuracy of echo parameters for the determinants of LAA thrombus in 140 persistent AF patients with a low CHADS2 score (≤1)

**Cutoff values**	**Sensitivity**	**Specificity**	**PPV**	**NPV**
LAAPV (≤ 24 cm/sec)	73 (66–80)	75 (68–82)	26 (18–33)	96 (92–99)
LAAEF (≤ 21%)	93 (89–97)	96 (92–99)	74 (67–81)	99 (97–100)

Data are percentages. Numbers in parentheses are 95% confidence intervals. PPV: positive predictive value. NPV: negative predictive value. Other abbreviations as in Table 1.

## Discussion

The present study demonstrated that LAAEF assessed by VVI was an independent determinant of LAA thrombus. Since the association between these parameters and LAA thrombus were independent of other clinical and echocardiographic parameters, LAAEF may be useful for thromboembolic risk stratification. AF patients with reduced LAAEF (≤ 21%) may require stronger anticoagulant therapy than patients with LAAEF >21% to avoid thromboembolic events, even if their CHADS2 score is low.

### LAA thrombus in patients with a low CHADS2 score

Thromboembolic events related to AF result in significant morbidity and mortality. However, the risk of thromboembolic events is quite variable [18-21]. A higher CHADS2 score [3-6] is associated with an increased risk of systemic embolism, bleeding and death in patients with AF receiving oral anticoagulants [22]. However, the CHADS2 score may not always be a good predictor of stroke risk, especially in intermediate risk patients and patients with relatively low scores [13]. A previous study reported that 10% of persistent AF patients had LAA thrombus confirmed by TEE prior to catheter ablation despite an average PT-INR of 1.9 ± 0.5, and 77% of the patients with LAA thrombus had a CHADS2 score ≤2 [23]. In the present study, LAA thrombus was found in 15/140 (10.7%) patients with a CHADS2 score ≤1. Among all of the patients, LAA thrombus was found in 16.5%. This may be due to the fact that target PT-INR level was achieved only in the two thirds of the patients. However, this high rate of LAA thrombus in the present study was concordant with that in a previous study (15.6%) [24]. Some patients with AF that receiving warfarin and had a CHADS2 score ≤1 suffered from cerebral embolism because warfarin was not totally effective in preventing LAA thrombus, despite a PT-INR in the target range. Therefore, better risk stratification is needed to improve the selection of AF patients who require strong anticoagulant therapy to prevent stroke.

TEE is known as the most sensitive and specific tool to assess LAA contractile fraction and detect LAA thrombus [17,23]. Daniel et al. suggested that TEE was associated with an acceptable low risk when performed by experienced operators under safe conditions [25]. In addition, we used a pediatric probe to minimize patient discomfort under safe conditions, as previously described [15]. Multivariate analysis in the present study showed that the association between LAA thrombus and LAA contractility was stronger than the association between LAA thrombus and age, hypertension and prior stroke. A recent TEE study demonstrated that increased LA volume and lower LV ejection fraction were significant predictors of LAA thrombus [24]. However, LAA motion was not considered in this recent study. The present study suggested that an LAAEF cutoff value of 21%, as measured by VVI echocardiography, should be used in a future prospective study to predict AF-related thrombus. Performing TEE in patients with a low CHADS2 score is controversial. However, our results suggest that evaluation of LAAEF by TEE is important, despite discomfort during the procedure because of the risk of cerebral infarction due to thromboembolism.

### Study limitations

There are several limitations of the present study. First, our findings are based on an observational study with a relatively small number of patients, particularly in the patients with both AF and LAA thrombus. We cannot draw conclusions regarding long-term outcomes, because the present study was a cross-sectional study. A prospective study in a lager patient population is needed to validate the predictive value of LAAEF and LAA peak strain on long-term outcomes. The predictive accuracy of the selected cutoff value for LAAEF and strain needs to be tested prospectively in an independent population to confirm its ability to predict LAA thrombus. Second, it is possible that the incidence of LAA thrombus does not always predict the occurrence of thromboembolic events because thromboembolism may be affected by other factors such as age, hypertension and diabetes mellitus. Finally, we measured the PT-INR levels one month before and at the time of TTE and TEE and averaged the two measurements. However, we recognized that there could be disparity between the measurements, and average value may not provide an accurate index of blood coagulability during this one-month period. In general, accurate evaluation of the blood coagulability was not possible.

## Conclusions

LAAEF in AF patients with thrombus was reduced compared with the values in those without thrombus, and this parameter was an independent determinant of LAA thrombus among various clinical and echocardiographic parameters, even in patients with a low CHADS2 score. AF patients with reduced LAAEF may require stronger anticoagulant therapy than patients with LAAEF >21% to avoid thromboembolic events, even if they have a low CHADS2 score.

## Abbreviations

LAA: Left atrial appendage; VVI: Velocity Vector Imaging; AF: Atrial fibrillation; LA: Left atrial; TTE: Transthoracic echocardiography; TEE: Transesopageal echocardiography; PT-INR: International normalized ratio of prothrombin time; LAAEF: Left atrial appendage emptying fraction; SEC: Spontaneous echo contrast; LAAPV: Left atrial appendage peak emptying flow velocity; LAD: Left atrial dimension; LV: Left ventricular; ROC: Receiver operating characteristic.

## Competing interests

The authors declare that they have no competing interests.

## Authors’ contributions

KO and MI carried out subject recruitment and analyzed data. MK and RT analyzed data and wrote the manuscript. TW, NO, SW, TK, TK, and YG performed ultrasound analysis. MA, KN, GT, TN, SW and SM analyzed data. All authors read and approved the final manuscript.
